# *Balamuthia mandrillaris* trophozoites ingest human neuronal cells via a trogocytosis-independent mechanism

**DOI:** 10.1186/s13071-022-05306-7

**Published:** 2022-06-27

**Authors:** Worakamol Pengsart, Nongnat Tongkrajang, Narisara Whangviboonkij, Patsharaporn Techasintana Sarasombath, Kasem Kulkeaw

**Affiliations:** 1 Faculty of Graduate Studies, Mahidol University, Nakhon Pathom, Thailand; 2 Siriraj Integrative Center for Neglected Parasitic Diseases, Department of Parasitology, Faculty of Medicine Siriraj Hospital, Mahidol University, 7th floor Adulyadejvikrom Building, 2 Wang Lang Road, Khwaeng Siriraj, Khet Bangkok-noi, Bangkok, 10700 Thailand

**Keywords:** *Balamuthia mandrillaris*, SH-SY5Y, Three-dimensional imaging, Trogocytosis

## Abstract

**Background:**

Environmental protozoa need an adaptation mechanism to survive drastic changes in niches in the human body. In the brain parenchyma, *Balamuthia mandrillaris* trophozoites, which are causative agents of fatal brain damage, must acquire nutrients through the ingestion of surrounding cells. However, the mechanism deployed by the trophozoites for cellular uptake remains unknown.

**Methods:**

Amoebic ingestion of human neural cell components was investigated using a coculture system of clinically isolated *B. mandrillaris* trophozoites and human neuroblastoma SH-SY5Y cells. Cell-to-cell interactions were visualized in a three-dimensional manner using confocal and holotomographic microscopes.

**Results:**

The *B. mandrillaris* trophozoites first attached themselves to human neuroblastoma SH-SY5Y cells and then twisted themselves around the cytoplasmic bridge. Based on fluorescence-based cell tracking, the *B. mandrillaris* trophozoites then inserted invadopodia into the cytoplasm of the human cells. Subsequently, the human protein-enriched components were internalized into the trophozoites in the form of nonmembranous granules, whereas the human lipids were dispersed in the cytoplasm. Intervention of trogocytosis, a process involving nibbling on parts of the target cells, failed to inhibit this cellular uptake.

**Conclusions:**

Human cell ingestion by *B. mandrillaris* trophozoites likely differs from trogocytosis, suggesting that a pathogen-specific strategy can be used to ameliorate brain damage.

**Graphical Abstract:**

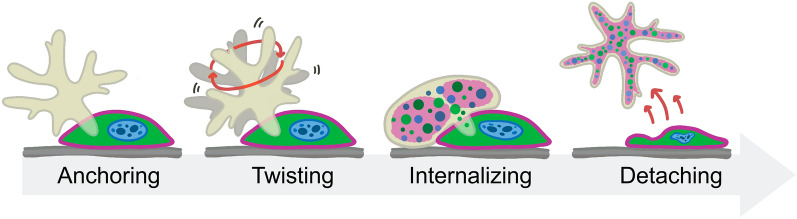

**Supplementary Information:**

The online version contains supplementary material available at 10.1186/s13071-022-05306-7.

## Background

The adaptability of single-cell organisms to different habitats plays a role in biological fitness. From the environment to the human body, biological niches change drastically, including via interactions with surrounding cells and different energy sources. Ingestion of human cells has been proposed as a direct process that parasitic protozoa deploy as an inside-host survival mechanism. Therefore, the sensing of host nutrients is a potential target for inhibiting pathogenesis, and has been extensively studied as a strategy to address parasite virulence [1].

*Balamuthia mandrillaris* is a natural nonsymbiotic amoeba that lives freely in soil and fresh water worldwide [2]. Despite being an environmental microorganism, *B. mandrillaris* is pathogenic to humans and can infect the brain, causing hemorrhagic necrosis known as granulomatous amoebic encephalitis (GAE) in the brain parenchyma. Although GAE is rare globally [3], it is a life-threatening disease in all age groups regardless of immune status [4]. The routes of transmission to humans include direct contact with soil via skin breakage, breathing dust of contaminated soil or through contact with fresh water through the nose. The current treatment strategies vary and rely on a combination of antibiotic or antifungal drugs [5–8]. Some regimens include drugs targeting *Leishmania* spp., a flagellate parasitic protozoan. Nevertheless, none of these strategies are effective, and thus the infection is associated with a high mortality rate (approx. 98%) [9]. To date, only six patients have recovered after treatment with the following drug regimen: pentamidine isethionate, 5-flucytosine, fluconazole and a macrolide (clarithromycin or azithromycin) with or without sulfadiazine, miltefosine, thioridazine or liposomal amphotericin B [10]. However, these drug combinations require long-term application, ranging from approximately months to years. Given its extreme rarity and difficult diagnosis, GAE is considered to be a neglected parasitic disease with a high mortality rate.

Attempts to develop new drugs have focused primarily on inhibiting the survival of the *B. mandrillaris* trophozoite, the vegetative stage. To examine amoebicidal activity, human cell-free culture of *B. mandrillaris* trophozoites is useful for high-throughput drug screening. However, given the ability of *B. mandrillaris* trophozoites to survive in the in vivo microenvironment, the use of human cell-free cultures does not allow the study of the process by which trophozoites acquire nutrients from the surrounding human cells. Studies have shown a cell contact-dependent mechanism in which *B. mandrillaris* trophozoites protrude into the cytoplasm to envelop and invade murine cells [11]. Dunnebacke [12] proposed a cell-in-cell formation of *B. mandrillaris* trophozoites in African green monkey kidney cells, followed by human cell death. Nevertheless, none of these studies demonstrated the cellular uptake of human cells, specifically, the questions of which cell components are ingested and in what form remain unaddressed. A recent study demonstrated that the epithelium-invading *Entamoeba histolytica* trophozoites nibbled parts of the human cell cytoplasm, a process defined as trogocytosis [13]. However, trogocytosis by *B. mandrillaris* trophozoites has not been reported to date. Altogether, the mechanism by which the *B. mandrillaris* trophozoite utilizes human neuron-related cells as an energy source for survival remains uncertain.

To obtain evidence of cellular uptake, in the present study we used a confocal microscope and a holotomographic microscope to image cell-to-cell interactions in a three-dimensional (3D) manner. To mimic human neurons, we used a conventional culture system of the human neuroblastoma SH-SY5Y cell line. Ingestion of human cell components was monitored using fluorescent probes specific to lipids, proteins and nucleic acids. Based on the results, we propose a trogocytosis-independent mechanism by which *B. mandrillaris* trophozoites take up cell components in standard 2D culture.

## Methods

### Culture of human neuroblastoma cells

The human neuroblastoma SH-SY5Y cell line (ATCC® No. CRL-2266™; ATCC, Manassas, VA, USA) was cultured in a mixture of ATCC-formulated Eagle's minimum essential medium (EMEM) and F12 medium (1:1 ratio) supplemented with 10% fetal bovine serum, hereinafter referred to as complete EMEM-F12, and incubated at 37 °C in a humidified atmosphere containing 5% CO_2_. Subculture was performed when cells reached 60–80% confluence following the ATCC’s instructions.

### Coculture of *B. mandrillaris* trophozoites with human neuroblastoma cells

*Balamuthia mandrillaris* trophozoites were cultured with human neuroblastoma SH-SY5Y cells using the same medium and conditions similar to those used for the culture of human neuroblastoma cells. Subculture of the amoeba was performed when > 90% of human neuroblastoma cells disappeared but prior to the formation of cysts (< 5% to enrich trophozoites). The floating trophozoites were collected and diluted with complete EMEM-F12 medium at a ratio of 1:5, followed by plating into a cell culture well containing human neuroblastoma cells at 80% confluence.

### Cell labeling with fluorescent probes

SH-SY5Y cells were cultured at a density of 2.5 × 10^5^ cells/250 μl medium in a well of a 48-well polystyrene plastic plate. At 72 h post culture, the medium was removed, and the cells were washed once with phosphate buffered saline (PBS). To label amines, the cells were incubated with the fluorescent dye CellTracker™ Green CMFDA (Invitrogen™, Thermo Fisher Scientific, Waltham, MA, USA OR), prepared at 2.5 μM in complete EMEM/F12 medium, for 45 min at 37 °C in a 5% CO_2_ incubator. For lipid binding, Vybrant™ DiD Cell-Labeling Solution (Invitrogen™, Thermo Fisher Scientific), a dye delivery system, was prepared at a 1:1600 dilution in complete EMEM-F12 medium and incubated with cells for 15 min as mentioned above. After labeling, the cells were washed 2–3 times with PBS to remove excess probes. SH-SY5Y cells labeled with *B. mandrillaris* trophozoites were cultured within 3 h following cell labeling.

### Confocal imaging of fluorescently labeled cells

To visualize cell–cell interactions in a snapshot manner, human neuroblastoma SH-SY5Y cells were allowed to adhere to a glass plate inserted into a well of a cell culture plate. At each time point, the culture medium was removed, and the cells were fixed with 2% paraformaldehyde (PFA) in PBS for 30 min at 37 °C. The glass plate was then lifted and placed upside down on a glass microscope slide. A drop of Fluoroshield Mounting Medium with DAPI (Abcam, Oxford, UK) was used for mounting the glass plate with adherent cells on the glass microscope slide. The cell-mounted microscope slides were kept in the dark prior to visualization under a confocal microscope (Nikon A1R; Nikon Corporation, Tokyo, Japan). For floating cells, the culture medium was harvested and centrifuged at 1200 rpm for 5 min at room temperature. After removal of the culture medium, the cell pellet was dispersed in polyvinyl alcohol as a fixative agent. A total of 20–25 µl of cell suspension was dropped and smeared on a glass microscope slide by circling the plastic tip in an outward direction. After air drying in the dark, the cell-mounted slide was subjected to microscopic imaging as mentioned above.

### Three-dimensional imaging

To observe cell-to-cell interactions in a 3D manner, cocultures of *B. mandrillaris* trophozoites with human SH-SY5Y cells were subjected to visualization under a holotomographic microscope (Tomocube Inc., Yuseong-gu, Daejeon, South Korea). When a light beam traverses a cell, each cellular compartment scatters light differently, generating an optical parameter known as the reflective index (RI). Hence, cells can be visualized based on the RI of the cellular compartments, allowing fluorescence-free imaging. Moreover, 360 ° rotation of the light source allows the construction of a holographic image of cell-to-cell interactions. Human neuroblastoma SH-SY5Y cells were cultured in a cell culture dish in a centered quadrilateral well (TomoDish, Daejeon, South Korea). The trophozoites were placed in the wells and subjected to 3D imaging. In some experiments, fluorescent probes were used to image the ingestion of human cells in real-time.

### Inhibition of trogocytosis

Wortmannin (Sigma-Aldrich, St. Louis, MO, USA), a phosphatidylinositol-3 kinase inhibitor, was incubated with the trophozoites following a previously protocol [13]. After removing the inhibitors, the trophozoites were cocultured with human neuroblastoma cells attached to a glass plate. Cells were observed using a confocal microscope as mentioned above.

### Cytotoxicity assays

Loss of cell membrane integrity was examined using trypan blue (Thermo Fisher Scientific), a conventional visible dye, and SYTOX™ Blue Dead Cell Stain, a molecular probe (Thermo Fisher Scientific). For trypan blue staining, the culture medium was removed, and PBS containing 0.4% trypan blue was added. After 1–2 min of incubation, the cells were washed with PBS and observed under an inverted microscope. For SYTOX Blue staining, human SH-SY5Y cells were plated on a glass slide placed in a well of a cell culture plate and incubated with *B. mandrillaris* trophozoites. SYTOX Blue was then added to the culture medium for 5 min, following which the cells were fixed to the glass using 4% PFA in PBS and mounted with an antifade medium (Fluoroshield Mounting Medium with DAPI; Abcam). To distinguish trophozoites and human cells, differential interference contrast or 5-chloromethylfluorescein diacetate (CMFDA) labeling was used to identify the cell shape of *B. mandrillaris* trophozoites. Cells were observed using a confocal microscope (Nikon AIR; Nikon Corporation).

### Quantitative PCR

Human neuroblastoma SH-SY5Y cells were subjected to RNA extraction using the FavorPrep™ Tissue Total RNA Extraction Mini Kit (Favorgen, Ping-Tung, Taiwan). The concentration and purity of the RNA were examined using a Nanodrop spectrophotometer (Thermo Fisher Scientific). The threshold for RNA purity was > 1.80. The mRNA was reverse transcribed to cDNA using the oligo-dT primer of iScript™ Reverse Transcription Supermix (Bio-Rad Laboratories, Hercules, CA, USA). The cDNA was used as a template to amplify B-cell lymphoma 2 (*BCL2*) and BCL2-associated X (*BAX*) transcripts. PCR was performed using Luna® Universal qPCR Master Mix (New England Biolabs, Ipswich, MA, USA) and the following primers: 5ʹ-TCATGTGTGTGGAGAGCGTC-3ʹ (forward primer) and 5ʹ-TCAGTCATCCACAGGGCGAT-3ʹ (reverse primer) for the *BCL2* transcript [14], and 5ʹ-TCAGGATGCGTCCACCAAGAAG-3ʹ (forward primer) and 5ʹ-TGTGTCCACGGCGGCAATCATC-3ʹ (reverse primer) for the *BAX* transcript [15]. Following a 3-min denaturation at 95 °C, DNA amplification included 40 rounds of denaturation at 95 °C for 15 s and annealing-extension at 60 °C for 30 s. The level of the human *ACTB* (Actin Beta) transcript was set as an internal control to normalize the levels of *BCL2* and *BAX* transcripts among samples. Threshold cycles of the samples were subjected to a calculation of relative gene expression based on the 2^−ΔΔCT^ method [16].

### Statistical analysis

The mean and standard deviation were calculated from at least three independent experiments. Graph plotting and statistical analysis were performed using GraphPad Prism 7 (GraphPad Inc., San Diego, CA, USA). Student’s t-test was used to compare the means of two different experimental settings, and a *p* value of < 0.05 was regarded as statistically significant.

## Results

**Fig. 1:**
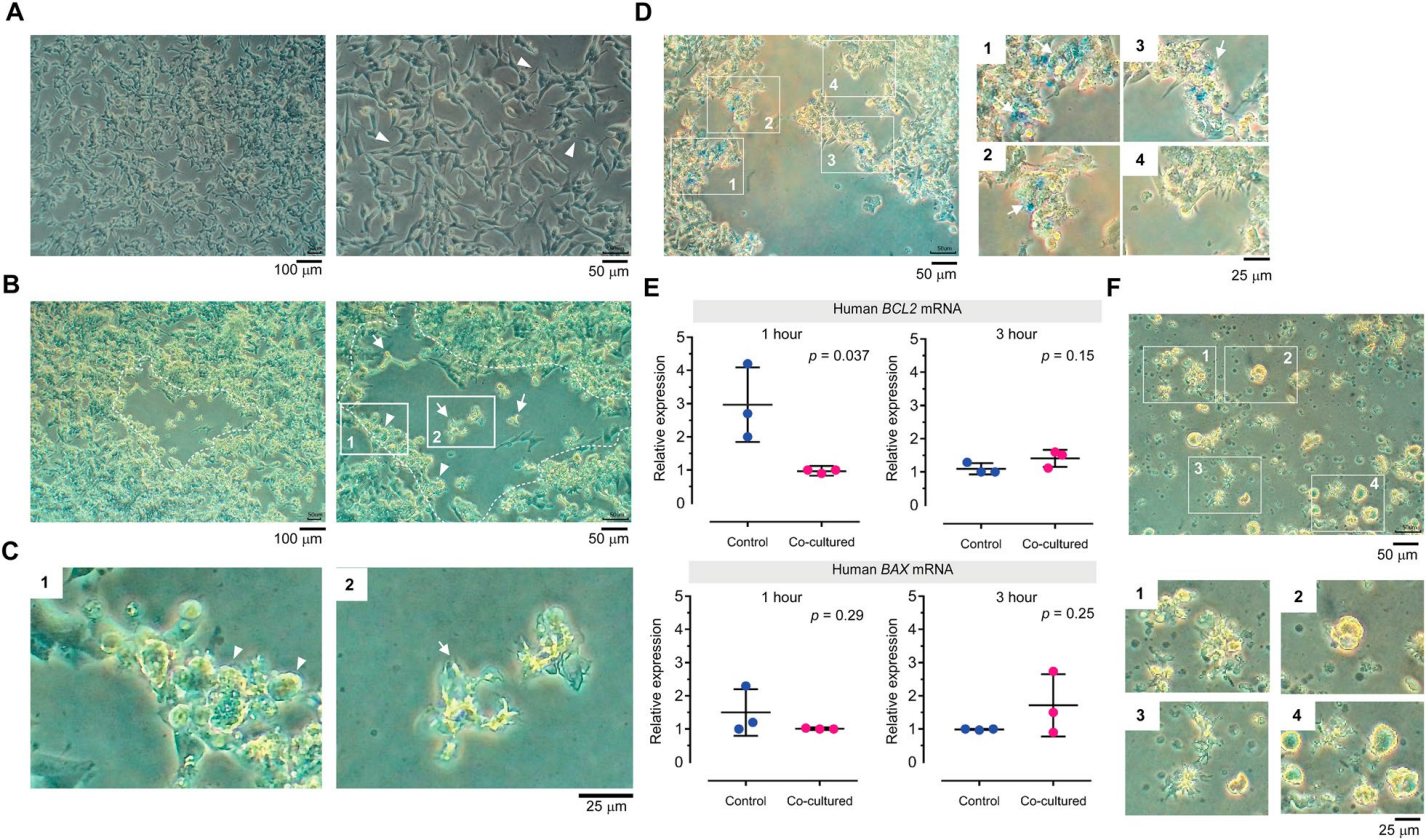
**A** Phase contrast image of human neuroblastoma SH-SY5Y cells under a light inverted microscope at 4× (left panel, scale bar: 100 μm) and 20× (right panel, scale bar: 50 μm) magnification. Arrowheads, neuroblast-like morphology with a few short neurite-like projections. **B** Microscopic images of the *Balamuthia mandrillaris* trophozoites cocultured with human neuroblastoma SH-SY5Y cells at 4× (left panel, scale bar: 100 μm) and 20× (right panel, scale bar: 50 μm) magnification. Dotted line, human cell-free area; Arrowheads, round shaped trophozoites; Arrows, cytoplasm-protruding trophozoites.  **C** Higher-magnification views of the insets in Fig. 1B. Scale bar: 25 μm. **D** Trypan blue-stained human neuroblastoma SH-SY5Y cells. Scale bar (left panel): 50 μm; scale bar (right panel): 25 μm. Arrows, trypan blue-positive human neuroblastoma cells. **E** Relative level of mRNA encoding human anti-apoptotic BCL2 and apoptotic BAX protein at 1 and 3 h post coculture. The levels are the mean ± SD of triplicate experiments. **F** Microscopic image of *B. mandrillaris* trophozoites on day 3 of coculture with the monolayer of human neuroblastoma SH-Sy5Y cells. Abbreviations: BAX, BCL2-associated X; BCL2, B-cell lymphoma 2; SD, standard deviation

In 2D culture, human neuroblastoma SH-SY5Y cells exhibit a neuroblast-like morphology with a few short neurite-like projections (arrowheads in the right panel of Fig. 1A). At 70–80% confluence, the trophozoites of *B. mandrillaris* were added to the culture medium of human neuroblastoma cells and plated on a monolayer of human neuroblastoma SH-SY5Y cells. Within 30 min, nearly all of the trophozoites became attached to the human cells. At 24 h post coculture, a human cell-free area appeared and increased in size in a time-dependent manner (the dotted line in Fig. 1B). At higher magnification, round cells could be seen at the edge of the human cell-free zone (arrowheads in inset #1 in the right panel of Fig. 1B and the left panel of Fig. 1C), while the irregularly shaped, cytoplasm-protruding trophozoites were located at the center of the human cell-free area (arrow in inset #2 in the right panel of Fig. 1B and the right panel of Fig. 1C). Live imaging of the inverted phase contrast microscope shows a trophozoite attached to human cells and protruding pseudopods. The human cell-adhering trophozoite also rotated, while its pseudopods occasionally elongated and shortened (Additional file 1: Video clip 1).

To examine the cytotoxicity of the *B. mandrillaris* trophozoites, we added live cell-impermeable trypan blue dye to the culture system at 24 h post coculture. The trypan blue-positive human neuroblastoma cells were proximal to the round trophozoites (inset #1–3 in Fig. 1D), indicating cell death due to loss of cell membrane integrity. In contrast, the trophozoite-free human cells were viable (inset #4 in the right panel of Fig. 1D). Downregulation of anti-apoptotic *BCL2* transcripts were observed at 1 h post coculture, while levels of apoptotic *BAX* transcripts tended to be upregulated but not significantly (Fig. 1E). After 2 days, the monolayer of human neuroblastoma cells was entirely destroyed (upper panel of Fig. 1F). Cell debris, pseudopod-protruding trophozoites and floating round-shaped cells were scattered throughout the culture well (insets #1–4, Fig. 1F). Taken together, these findings show that the clinically isolated *B. mandrillaris* trophozoites were cytotoxic to human neuroblastoma SH-SY5Y cells.

**Fig. 2:**
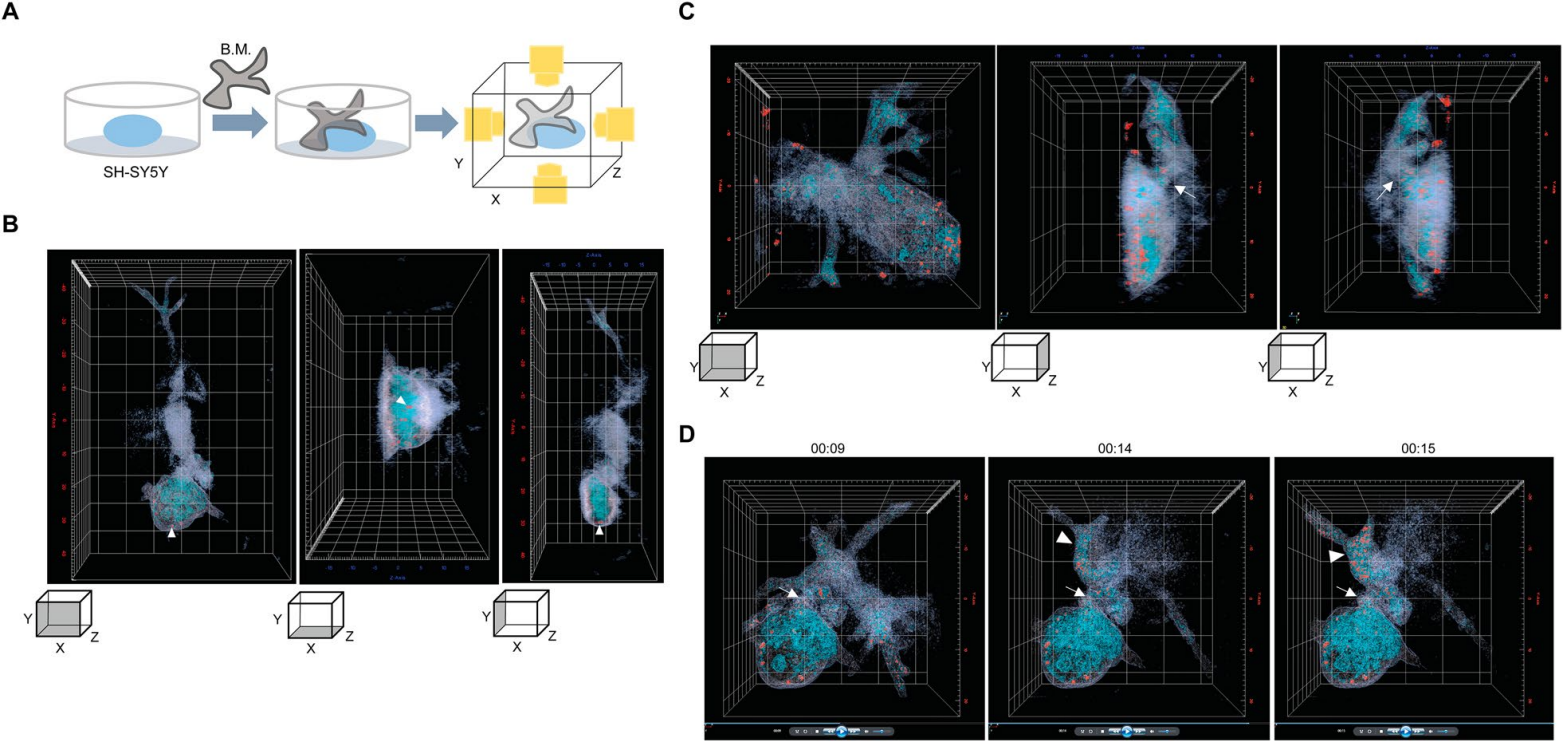
**A** Schematic diagram of 3D imaging of cell-to-cell interactions using a holotomographic microscope. **B** The 3D images of pseudo-colored cells along the X–Y, X–Z and Y–Z planes are shown in the panels from left to right. Arrowheads, lipid droplet-like dots. **C** X–Y view of the *B. mandrillaris* trophozoites interacting with human neuroblastoma SH-SY5Y cells (left panel), and the cytoplasmic bridge (arrows in the middle and right panel) in the Y–Z plane. **D** The snapshot image of a rotating trophozoite shows anchoring sites (arrows in the left, middle and right panels). Abbreviations: B.M., *B. mandrillaris*; SH-SY5Y, human neuroblastoma cell line

To observe the mechanism by which the trophozoites ingest human cellular components in real time, we used a holotomographic microscope to capture 360 ° views of the 2D-cultured cells (Fig. 2A). The 2D view of cells in the X–Y plane showed a difference in intensity from high to low (white to black; Additional file 2: Fig. S1). The dense white-colored circles observed in the 2D image of human cells were lipid droplet-like structures (arrowhead in Additional file 2: Fig. S1). Based on morphology, the white oval shape was distinct from the elongated shape with protruding pseudopods. However, without these differences in cell morphology, it was difficult to distinguish between the human cells and trophozoites in the 2D image (Additional file 2: Fig. S1A).

Given that different types of cell components exhibit distinct refractive indexes (RIs), the holotomographic microscope is capable of visualizing cell components in a 3D manner. Based on the RIs of the cell components, pseudocolors were assigned for each cell component. As shown in the X–Y plane (left panel in Fig. 2B), the cytoplasm of human neuronal cells exhibited a cyan color, while that of trophozoites displayed a blue gray color. Red dots that had an RI similar to that of the lipid droplets were observed along the X–Y, X–Z and Y–Z planes (arrowheads in all panels of Fig. 2B; Additional file 3: Video clip 2). For the second representative cell image, the X–Y plane showed oval-shaped cells with a protruding pseudopod-like structure. When observing the lateral view along the Y–Z planes, there was a bridge between the human neuroblastoma cells and *B. mandrillaris* trophozoites (arrows in the middle and left panels of Fig. 2C and Additional file 4: Video clip 3). Thus, the difference in the RI between human neuroblastoma cells and *B. mandrillaris* trophozoites allows the identification of the cell type and connecting point.

To observe the movement of the trophozoites, live imaging was performed using holotomography to observe cell-to-cell interactions. The 3D live imaging revealed that the *B. mandrillaris* trophozoite twisted its extending pseudopod (Additional file 5: Video clip 4). When trophozoite rotation was imaged for 6 s, the snapshot image shows anchoring sites (arrows in the all panels of Fig. 2D). Notably, the number of cyan-colored components increased in the pseudopod-protruding trophozoite when observed for 6 s (arrowhead in the middle and right panels of Fig. 2D). Taken together, these results show that the *B. mandrillaris* trophozoite anchors to the human cell, then twists its extending cytoplasm around the cytoplasmic bridge.

**Fig. 3:**
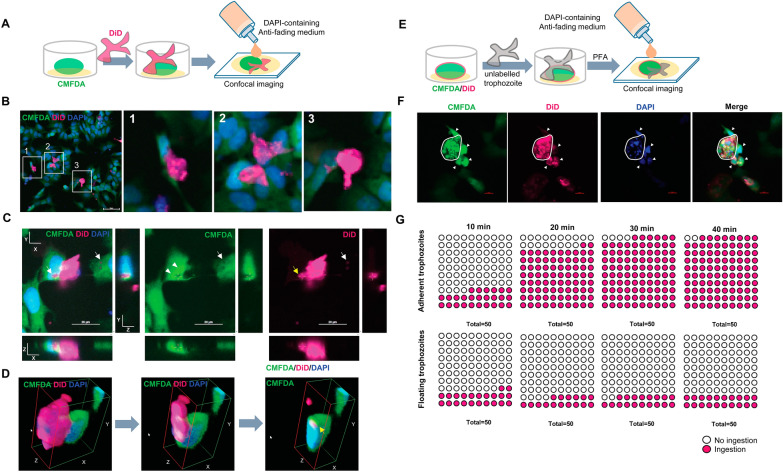
**A** Schematic diagram of the differential cell tracking of CMFDA-labeled human neuroblastoma cells (green) and DiD-labeled *B. mandrillaris* trophozoites (magenta), followed by coculture. **B** Cell-to-cell interactions of the DiD-labeled trophozoites (magenta) with human SH-SY5Y cells (green for CMFDA and blue for DAPI) at 40 min post coculture. **C** Invadopodia of the *B. mandrillaris* trophozoites protruding into human neuroblastoma cells. Arrows, the cytoplasm of human cells; Yellow arrow, invadopodia-like structure.  **D** The X–Y-Z plane of confocal images of the DiD-labeled trophozoites (magenta) with human SH-SY5Y cells (green for CMFDA and blue for DAPI). Yellow arrow, invadopodia-like structure. **E** Schematic diagram of the cellular uptake of unlabeled *B. mandrillaris* trophozoites. **F** Confocal images of the *B. mandrillaris* trophozoites along the X–Y plane. Dotted line, the cytoplasm of the *B. mandrillaris* trophozoites; Arrowheads, human nuclei.  **G** Proportion of *B. mandrillaris* trophozoites that ingested human cell components (magenta circles). Abbreviations: DAPI, 4′,6-Diamidino-2-phenylindole; DiD, 1, 1-dioctadecyl-3,3,3,3-tetramethylindodicarbocyanine; PCMFA, 5-chloromethylfluorescein diacetate; PFA, paraformaldehyde

Given the similarity between the RIs of the human cells and the parasite, it was difficult to observe the invasion of the *B. mandrillaris* trophozoites. Thus, differential cell tracking was performed to observe a cell-to-cell interaction. Two fluorescence probes, CMFDA and 1, 1-dioctadecyl-3,3,3,3-tetramethylindodicarbocyanine (DiD), were used to label amines and lipids, respectively (Fig. 3A). In human neuroblastoma SH-SY5Y cells, CMFDA was observed as a smear pattern dispersed throughout the cytoplasm, while DiD was observed as a granule-like pattern in the cytoplasm and on the cell membrane (Additional file 6: Fig. S2A). In contrast, the cytoplasm of *B. mandrillaris* trophozoites exhibited a dispersed pattern of DiD-labeled lipids. There was no cellular component labeled with CMFDA (Additional ffile 6: Fig. S2B). These results indicated that the CMFDA and DiD fluorescent probes could be used for tracking human neuroblastoma cells and *B. mandrillaris* trophozoites, respectively (Additional file 6: Fig. S2C).

Based on light microscopic observations, Dunnebacke [12] reported that whole trophozoites entered African green monkey kidney cells. To confirm the cell-in-cell formation, CMFDA and DiD were used to label the respective human cells and the *B. mandrillaris* trophozoites to detect human cell-invading *B. mandrillaris* trophozoites (Fig. 3A). After 40 min of coculture on a glass slide, the X–Y plane of the 2D-captured image showed DiD-labeled *B. mandrillaris* trophozoites (magenta, Fig. 3B) located proximal to the CMFDA-labeled human neuronal cells (green, Fig. 3B). The trophozoites were positioned at the top of human neuroblastoma cells. Some trophozoites were partially adhered to the human cells (insets 1 and 3 of Fig. 3B), while some spread their cytoplasm across a few human cells (inset 2 of Fig. 3B). There were no yellow-colored merged areas, suggesting that none of the trophozoites entered the human cells.

To observe the invasion by *B. mandrillaris* trophozoites into human neuroblastoma cells, differential cell tracking was performed as mentioned above (Fig. 3A). At 40 min post coculture, magenta-colored holes were observed in the cytoplasm of human cells (arrows in all panels of Fig. 3C), indicating the presence of DiD-labeled lipids in *B. mandrillaris* trophozoites. By using the Z-stack, the X–Z and Y–Z planes allow a lateral view of the imaged cells (left panel of Fig. 1C). Thus, both the X–Z and Y–Z planes revealed that the DiD-labeled compartment was surrounded by the human cytoplasm (green) and was proximal to the human nucleus (left panel of Fig. 3C). The intensity of CMFDA fluorescence was higher around the cytoplasmic hole than in other parts of the human cytoplasm (arrowheads in middle panel of Fig. 3C). The lipid-containing cytoplasm of the *B. mandrillaris* trophozoite protruded into human cells, a structure mimicking invadopodia (yellow arrows in right panel of Fig. 3C). A 3D view of the X–Y-Z plane shows the invadopodia-like structure of *B. mandrillaris* trophozoites protruding into human neuroblastoma cells (yellow arrow in Fig. 3D; Additional file 7: Video clip 5).

Following invasion, the cellular uptake of the *B. mandrillaris* trophozoites was examined. To observe components of human cells inside the trophozoites, both CMFDA and DiD fluorescence probes were preincubated with human neuroblastoma cells, while the *B. mandrillaris* trophozoites remained unstained (Fig. 3E). In the cytoplasm of the *B. mandrillaris* trophozoites (dotted line in Fig. 3F), the amine-containing cell components appeared as well-defined granules with irregular sizes. In contrast, the lipid components were nonuniformly dispersed throughout the cytoplasm of *B. mandrillaris* trophozoites (Fig. 3F). The pattern of human-derived lipids in the cytoplasm of the trophozoites was similar to that of *B. mandrillaris*-derived lipids (Additional file 6: Fig. S2B). The majority of amines and lipids showed no overlap, indicating the existence of a distinct cellular compartment (merge panel of Fig. 3F). Moreover, the trophozoite contained sparse granular nuclei that were smaller than human nuclei (arrowheads in Fig. 3F). When the cellular uptake was examined, the percentages of CMFDA- and DiD-positive trophozoites adhering to human cells increased to 96% at 40 min post coculture (Fig. 3G). However, there were no changes in the proportion of human cell-ingesting floating trophozoites between 10 and 40 min post coculture. After 6 h of coculture, the *B. mandrillaris* trophozoites were mostly detached and floated in the culture medium. The floating trophozoites had ingested the human cell components, which appeared as amine-containing, irregularly shaped granules and lipid-diffused areas in the trophozoite cytoplasm (Additional file 8: Fig. S3). Altogether, these results suggest that the *B. mandrillaris* trophozoite protrudes its cytoplasm into human cells, forming a structure mimicking invadopodia, prior to ingesting human cell components.

**Fig. 4:**
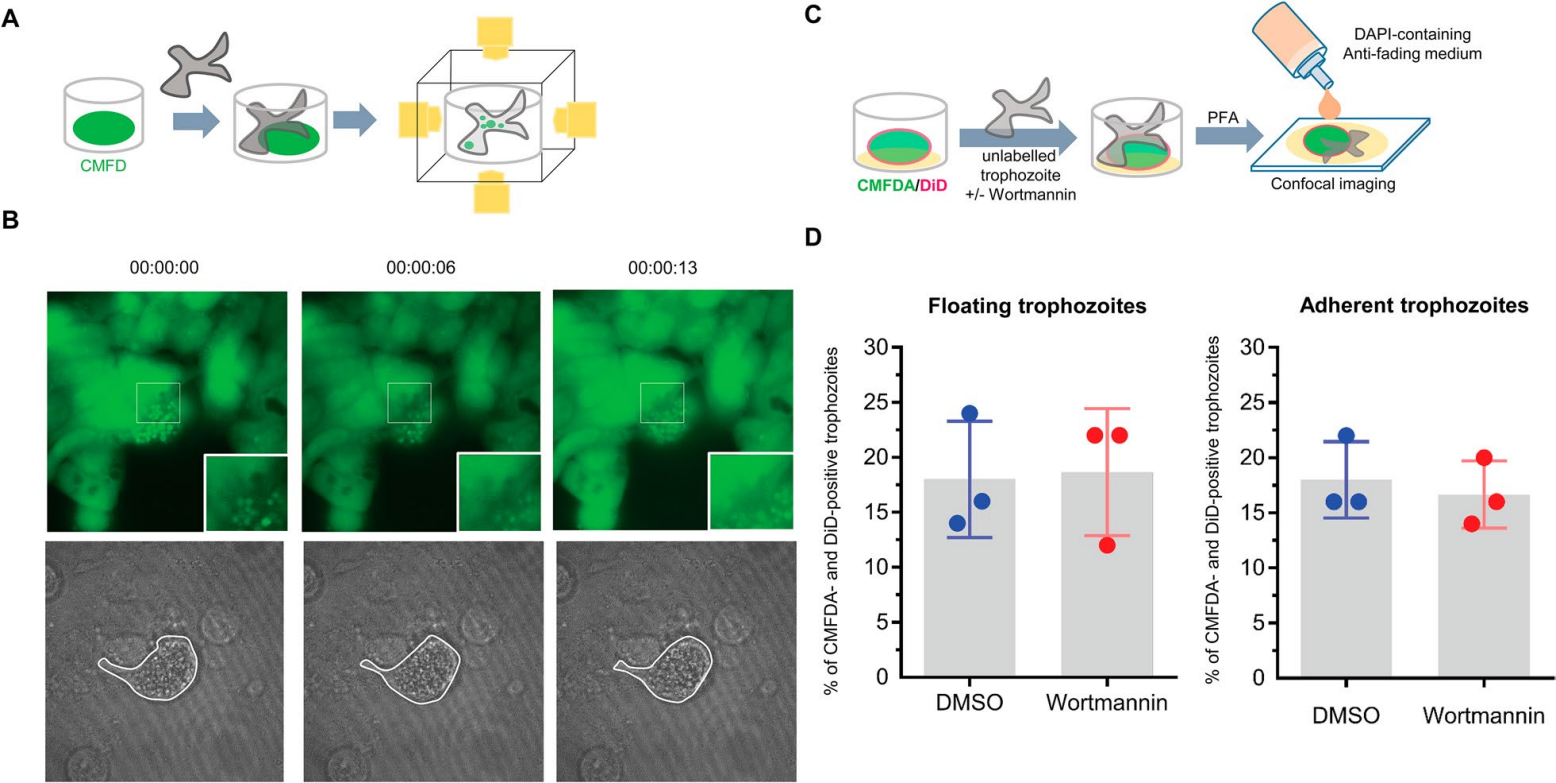
**A** Live imaging of CMFDA-labeled human neuroblastoma SH-SY5Y cells (green) cocultured with nonlabeled *B. mandrillaris* trophozoites using a holotomographic microscope. **B** Snapshot images of CMFDA-labeled human neuroblastoma SH-Sy5Y cells (green). Insets show higher-magnification images captured with a 60× objective lens. Dotted lines indicate *B. mandrillaris* trophozoites. **C** Schematic diagram of the cellular uptake of unlabeled *B. mandrillaris* trophozoites. **D** Wortmannin-mediated inhibition of trogocytosis and cell ingestion by *B. mandrillaris* trophozoites. Abbreviations: CMFDA, 5-Chloromethylfluorescein diacetate; DMSO, dimethyl sulfoxide

To visualize ingestion of the human cell cytoplasm in real time, the human cells were cultured as a 2D monolayer and incubated with the amine-binding fluorescent dye CMFDA (Fig. 4A. The nonlabeled trophozoites were cocultured with CMFDA-labeled human cells. At 6 h post coculture, sparse green circular shapes were observed in the cytoplasm of the trophozoites. Live imaging showed that green vesicles appeared in the area proximal to the human cells (Additional file 9: Video clip 6). Within 13 min, the size of the colored area in the human cytoplasm increased, suggesting loss of the cytoplasmic compartment (inlets in Fig. 4B). Subsequently, the human cells lost cell membrane integrity, likely due to apoptosis (Fig. 4C). Thus, the *B. mandrillaris* trophozoites ingested the human protein constituent in the form of spherical granules, a finding similar to the 2D snapshot confocal images in Fig. 3F.

The phosphatidylinositol-3 kinase (PI3K) pathway plays an important role in trogocytosis, a process in which parasitic amoebae ingest portions of human cells [13]. To investigate whether human cell ingestion by *B. mandrillaris* relies on the PI3K signal, human trophozoites were preincubated with wortmannin, a PI3K inhibitor, prior to 40-min-long coculture with CMFDA-labeled human cells (Fig. 4C). The number of CMFDA- and DiD-positive trophozoites indicated that the *B. mandrillaris* trophozoites incubated with wortmannin retained the capability of ingesting human amine-containing components, demonstrating that ingestion by *B. mandrillaris* trophozoites was trogocytosis independent (Fig. 4D).

## Discussion

In the present study, the mechanism underlying *B. mandrillaris*-induced cellular damage was examined in a standard 2D culture of human neuroblastoma cells. Based on the 3D live imaging results, an anchoring-to-twisting behavior of the *B. mandrillaris* trophozoites was proposed. The 3D view of the confocal images revealed that the invadopodia first penetrate human cells, following which the trophozoites ingest human cell contents in two different forms: (i) protein-containing, nonmembranous droplets; and (ii) lipid-dispersed patterns. Inhibition of cell nibbling or trogocytosis failed to suppress these processes. Thus, the mechanism of cytopathogenicity of *B. mandrillaris* trophozoites is likely different from trogocytosis.

Despite the long-known effects of *B. mandrillaris* trophozoites on host cell death [17], the cellular uptake of pathogenic *B. mandrillaris* remains largely unaddressed. The number of studies conducted on the cytopathogenicity of *B. mandrillaris* to date is limited [11, 12]. Kiderlen et al. used murine mastocytoma P815 cells, an adherent phagocytic cell line derived from mast cells, to study the cytopathogenicity of a mandrill baboon-derived *B. mandrillaris* isolate [11]. These authors performed scanning electron microscopy and transmission electron microscopy studies and observed the engulfment and cytoplasmic protrusion of *B. mandrillaris* trophozoites, leading to murine cell lysis. Consequently, the cytopathogenicity of *B. mandrillaris* has been attributed to phagocytosis. Based on phase contrast images, Dunnebacke [12] reported that *B. mandrillaris* entered the cytoplasm of African green monkey kidney cells. In that study, 2D cultures of mouse and monkey-derived cells enabled cell-to-cell interactions to be observed mainly in a 2D manner.

Similar to previous studies, in the present study we used the 3D view of a confocal image for visualizing invadopodia. However, the 3D confocal images did not show internalization of the *B. mandrillaris* trophozoite into human cells, a cell-in-cell formation process [12]. The discrepancy between our results and those of Dunnebacke [12] may result from the host cell types, the amoeba strains and observation methods. Nevertheless, the use of a confocal microscope is more practical for laboratories equipped with a 3D fluorescence microscope. Advances in high-resolution imaging may support the quantitative analysis of confocal images, limiting subjective interpretation. Moreover, in the present study, live microscopic observation showed the position of the *B. mandrillaris* trophozoite to be on the top of or beside human neuroblastoma cells during cellular uptake. However, the holotomographic imaging in the current study also has limitations. First, the experiment was performed in a short period of 3 h due to the lack of a CO_2_ incubator with the holotomographic microscope. Second, the fluorescence intensity of CMFDA was diminished after light exposure, a process known as photobleaching. Thus, cell tracking with a more light-resistant fluorescent dye in a closed system will allow long-term observation under physiologically relevant conditions in real time.

Trogocytosis is a process by which parts of the target cell membrane are cut and internalized. It was first demonstrated in *Naegleria fowleri*, a parasitic amoeba causing primary amoebic meningoencephalitis [19]. Immune cells use trogocytosis for cell–cell communication, with the nibbling immune cells displaying membranous proteins of other immune cells to trigger defensive mechanisms [20–23]. Neutrophils also use trogocytosis to kill cancer cells and the urogenital tract-dwelling *Trichomonas vaginalis*. On the other hand, *E. histolytica* trophozoites, which are obligate parasitic amoebae, reportedly use trogocytosis to acquire the lipid-membrane-bound protein of human Jurkat T cells [13]. In addition to using this protein as an energy source, *E. histolytica* trophozoites also display this human membranous protein as a strategy to disguise themselves from host immune cells [24], a process known as cross-dressing. Whether *B. mandrillaris* trophozoites utilize human membrane proteins to escape the innate immune defense of the host needs to be further investigated.

The data acquired in this study indicate the trogocytosis-independent process to be a strategy to gain nutrients from human cells. Nevertheless, the reliability of some supporting evidence and limitations of the methods used should be considered. By using protein- and lipid-binding fluorescent dyes for 3D confocal microscopy, the *B. mandrillaris* trophozoites were found to take up human proteins in vesicle-like structures that lacked a phospholipid membrane. Thus, the process by which parts of ingested target cells are surrounded by phospholipid membranes is unlikely to be endocytosis or trogocytosis. In contrast, the human lipid components were dispersed nonuniformly throughout the cytoplasm of the *B. mandrillaris* trophozoites, indicating different ways for the transfer of lipids and proteins. Second, Rolstone et al. [13] clearly demonstrated that *E. histolytica* trophozoites take up lipid membrane-bound protein-containing vesicles from human leukemic T cells. Pretreatment with the PI3K inhibitor wortmannin suppressed the trogocytosis of *E. histolytica*. In contrast, interruption of the PI3K signal failed to inhibit the cellular uptake of *B. mandrillaris* trophozoites. Notably, the use of adherent and floating human cells to demonstrate cellular uptake may result in different interpretations of cellular uptake. Floating cancer cells take up particles in a phagocytosis-like manner at levels higher than that observed with adherent cancer cells [25]. Thus, an investigation of cellular uptake should be performed in a more physiologically relevant setting, i.e. cerebral organoid, neural spheroid or ex vivo culture, of human brain tissue [26].

CMFDA and DiD are fluorescent dyes that bind to amines and lipids, respectively. Human neuroblastoma cells exhibited a CMFDA-labeled cytoplasm, which contained protein, and a DiD-labeled phospholipid bilayer of the cell membrane. Moreover, labeling of the *B. mandrillaris* trophozoites using CMFDA and DiD showed a similar pattern to that of human neuroblastoma cells: CMFDA in the cytoplasm and DiD on the cell membrane. Therefore, the use of both cell tracking dyes is a reliable method to track cell components. However, there were limitations to the 3D imaging in the present study. Confocal microscopy allows laser-based 3D imaging of cells in a fixed form at a certain time point. Given the dynamic process of cellular uptake, live imaging would enable the observation of cell physiological phenomena in a relatively real-time manner. Thus, a holotomographic microscope was used to observe cellular uptake. It should be noted, however, that the holotomographic microscope used in this study allowed only the visualization of CMFDA.

To confirm the presence of invadopodia and occurrence of endocytosis, additional experiments in which these processes are inhibited using small-molecule inhibitors are needed. Wortmannin, an inhibitor of the PI3K signaling pathway, was able to inhibit amoebic trogocytosis of *E. histolytica* trophozoites despite a lack of data supporting defects in PISK signaling. Moreover, wortmannin-mediated PI3K inhibition of *Dictyostelium discoideum*, a single- or multiple-cell organism in the phylum amoebozoa, decreased the formation of bacteria-containing phagosomes. Thus, actin polymerization-mediated trogocytosis is an unlikely mechanism used by *B. mandrillaris*. To our knowledge, there are no assays to validate the effectiveness of PI3K inhibition in the trophozoites of *B. mandrillaris*, limiting the assessment of wortmannin’s effect in our experiments. Nevertheless, pretreating *B. mandrillaris* trophozoites with wortmannin did not inhibit cellular uptake.

*B. mandrillaris* trophozoites have been cocultured with feeder cells derived from human or nonhuman origin. Studies with African green monkey kidney cells, also known as Vero cells, supported the growth of *B. mandrillaris* isolated from the autopsied brain of a pregnant baboon (*Papio sphinx*) that died from meningoencephalitis, whereas human lung carcinoma MRC-5 cells failed to do so [18]. To our knowledge, human lung carcinoma A549 cells supported the growth of *B. mandrillaris* isolated from the autopsied brain of a Thai patient. In contrast, the use of human neuroblastoma SH-SY5Y cells showed that the Thai *Acanthamoeba* isolate caused human cell death via downregulation of apoptotic *BAX* gene expression. In this view, the trophozoites ingest and damage these cells regardless of the type, leading to cell death and removal of host cells from culture. Despite severe damage to feeder cells, the mechanisms of cytopathogenicity have not been investigated.

Given the physiological relevance to mature neurons, the human neuroblastoma SH-SY5Y cell line was used as a model for studies of neurotropic pathogens, neurodegenerative disease and neuronal differentiation. Most fatal clinical manifestations of *Balamuthia* encephalitis are caused by neuropathogenesis, including cell death in the brain parenchyma. Thus, SH-SY5Y is more relevant to human physiology than other cell lines. Notably, human SH-SY5Y cells can be induced to differentiate into mature neurons [27], allowing the screening of cytotoxicity in a high-throughput manner with more physiological relevance than undifferentiated cells [28].

Although the conventional 2D culture of human cancer cell lines is useful for studying host–parasite interactions, the current in vitro models have gradually shifted to a more physiologically relevant 3D setting. Spheroids and organoids are 3D culture systems in which cells are organized in a cluster, thereby allowing intercellular interactions in all dimensions and gradient exposure to external stimuli. The 2D-cultured cells differ from those of the 3D culture, including equal exposure to nutrients and gas, cell–cell interactions and cell-rigid surface interactions. As a result, the results on several biological processes of cells were not similar between the 2D and 3D studies. Spheroids of human neuroblastoma SH-SY5Y cells have been found to be relatively more mature than the 2D cell culture [29]. Therefore, the use of spheroids may address the gap in our understanding of the cytopathogenicity of *B. mandrillaris*.

## Conclusion

A clinical isolate of *B. mandrillaris* trophozoites is cytotoxic to human neuroblastoma SH-SY5Y cells. The 3D imaging performed in the present study revealed the anchoring-to-twisting behavior of the *B. mandrillaris* trophozoites. The addition of a Z-plane during confocal observation allowed visualization of invadopodia penetrating the human cytoplasm. There were at least two different types of cellular uptake: (i) protein-containing, nonmembranous, well-defined granules; and (ii) dispersed smears of lipid components. Inhibition of PI3K-mediated trogocytosis failed to suppress the cellular uptake of *B. mandrillaris* trophozoites, suggesting the existence of trogocytosis-independent cytopathogenicity.

## Supplementary Information

**Additional file 1: Video clip 1.** Live imaging of *B. mandrillaris *trophozoites in the 2D culture of human neuroblastoma SH-SY5Y cells.

**Additional file 2: Figure S1.** The 2D view (X-Y plane) of a holotomographic image of a *B. mandrillaris* trophozoite interacting with a human neuroblastoma cell. Scale bar = 10 μm.

**Additional file 3: Video clip 2. **A 3D image of* B. mandrillaris* trophozoites interacting with human neuroblastoma SH-SY5Y cells under a holotomographic microscope.

**Additional file 4: Video clip 3. **A 3D image of* B. mandrillaris *trophozoites interacting with human neuroblastoma SH-SY5Y cells under a holotomographic microscope. 

**Additional file 5: Video clip 4. **Live imaging of cell-to-cell interactions in the 3D image from the holotomographic microscope.

**Additional file 6: Figure S2. **Fluorescence-based cell tracking of human neuroblastoma SH-SY5Y cells and *B. mandrillaris *trophozoites. Labeling of human neuroblastoma SH-SY5Y cells with CMFDA (green) and DiD (magenta) prior to confocal imaging (a). Scale bars: 10 μm. A procedure for CMFDA and DiD labeling of *B. mandrillaris* trophozoites collected from a human cell-free culture (b). Scale bars: 10 μm. A sequential step in differential cell tracking of *B. mandrillaris* trophozoites and human neuroblastoma SH-SY5Y cells (c). Scale bars: 50 μm.

**Additional file 7: Video clip 5. **The 3D image of *B. mandrillaris *trophozoites (magenta) interacting with human neuroblastoma SH-SY5Y cells (green) captured by a confocal microscope. 

**Additional file 8: Figure S3. **Procedure to visualize human cell component-ingesting* B. mandrillaris* trophozoites that were not adherent to human cells. 

**Additional file 9: Video clip 6. **Live imaging of CMFDA-labeled human neuroblastoma SH-SY5Y cells (green) cocultured with nonlabeled *B. ma«ndrillaris* trophozoites under a holotomographic microscope.  

## Data Availability

All data generated or analyzed during this study are included in this published article and its Additional information files.

## Abbreviations

BAX: BCL2-associated X
BCL2: B-cell lymphoma 2
CMFDA: 5-Chloromethylfluorescein diacetate
DiD: 1, 1-Dioctadecyl-3,3,3,3-tetramethylindodicarbocyanine
EMEM: Eagle’s minimum essential medium
GAE: Granulomatous amoebic encephalitis
PBS: Phosphate-buffered saline
PI3K: Phosphoinositide 3-kinase
q-PCR: quantitative PCR
