# Supplementary figures and images for: Balamuthia mandrillaris trophozoites ingest human neuronal cells via a trogocytosis-independent mechanism
Source: Parasit Vectors. 2022 Jun 27;15:232. doi: 10.1186/s13071-022-05306-7 (PMC9235117; doi:10.1186/s13071-022-05306-7)

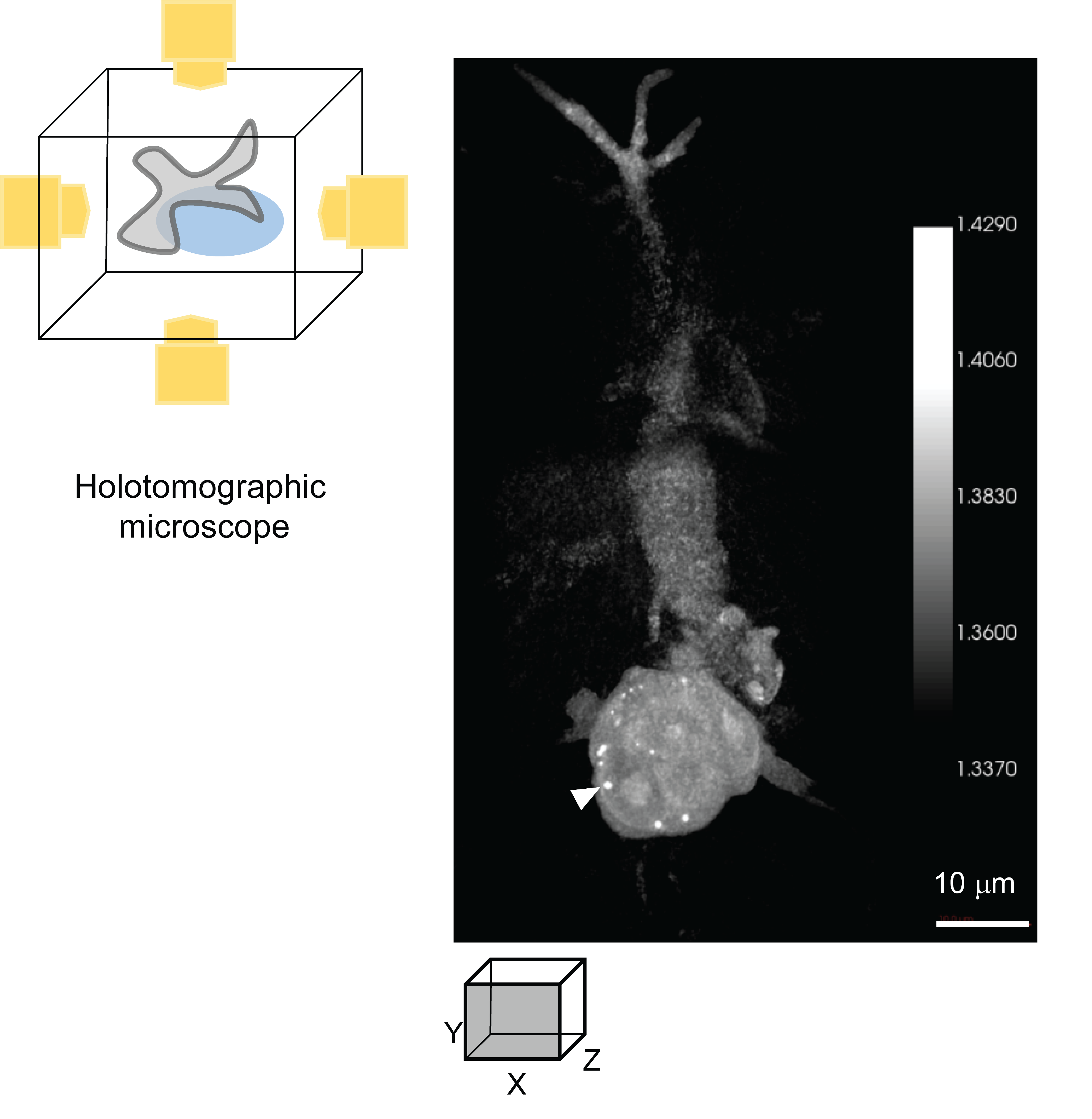

Supplement: Supplementary file 2 — Additional file 2: Figure S1. The 2D view (X-Y plane) of a holotomographic image of a B. mandrillaris trophozoite interacting with a human neuroblastoma cell. Scale bar = 10 μm. [file 13071_2022_5306_MOESM2_ESM.tif]
